# Association of polymorphisms of interleukin-18 gene promoter region with polycystic ovary syndrome in chinese population

**DOI:** 10.1186/1477-7827-8-125

**Published:** 2010-10-22

**Authors:** Yan Yang, Jie Qiao, Mei-zhi Li

**Affiliations:** 1 Department of Obstetrics and Gynecology, Peking University Third Hospital, Beijing, P.R China

## Abstract

**Background:**

Recent research shows that polycystic ovary syndrome (PCOS) may have an association with low-grade chronic inflammation, and that PCOS may induce an increase in serum interleukin-18 (IL-18) levels.

**Methods:**

To investigate the polymorphisms of the IL-18 gene promoters with PCOS, two single nucleotide polymorphisms (SNPs) in the promoter of the IL-18 gene (at positions -607C/A and -137G/C) in 118 Chinese women with PCOS and 79 controls were evaluated using polymerase chain reaction (PCR).

**Results:**

No significant differences were found in the genotype distribution, allele frequency and haplotype frequency between the PCOS and control groups. Further analysis demonstrated a relationship between IL-18 gene promoter polymorphisms and PCOS insulin resistance (IR). Regarding the -137 allele frequency, G and C allele frequencies were 93.5% and 6.5%, respectively, in the PCOS with IR patients; G and C allele frequencies were 85.4% and 14.6%, respectively, in PCOS patients without IR (chi2 = 3.601, P = 0.048).

**Conclusions:**

The presence of a polymorphism in the IL-18 gene was found to have no correlation with the occurrence of PCOS. Carriage of the C allele at position -137 in the promoter of the IL-18 gene may play a protective role from the development of PCOS IR.

## Background

Polycystic ovary syndrome (PCOS) is a common and complex endocrine disorder of women in their reproductive years, with prevalence between 5% and 10% of the general population [1,2]. The etiology of PCOS remains controversial, but there is evidence suggesting that genetic factors may play an important role [3,4]. Studies have shown that a common polymorphism of the interleukin-1α gene (IL) is associated with the presence of PCOS [5], and a polymorphism of the IL-6 promoter is associated with clinical characteristics of women affected by PCOS [6].

Three single nucleotide polymorphisms (SNPs) in the promoter of the IL-18 gene at positions -656G/T, -607C/A and -137G/C have been identified; the two SNPs at positions -607C/A and -137G/C are predicted to influence the expression of IL-18 and, potentially, interferon -γ (IFN)[7].

In order to investigate the possible roles of IL-18 in the pathogenesis of PCOS and its relationship with insulin resistance (IR), obesity and hyperandrogenism, the polymorphisms at positions -607 and -137 in the promoter of the IL-18 gene in PCOS patients has been investigated.

## Methods

### Patients and control groups

A total of 118 patients with PCOS were included in this prospective, case-controlled study. Mean age at diagnosis was 28.60 years (S.D. = 3.45). For this study, data from women with PCOS visiting the Department of Obstetrics and Gynecology, Division of Reproductive Center, Peking University Third Hospital, Beijing, from October 2006 to January 2007 were analyzed. All patients were Chinese and had not taken medications, including contraceptive pills, for the last 6 months. None of the patients had any clinical evidence (history or examination) of a recent or ongoing infection. Women having received any hormonal treatment or insulin-lowering agent during the last 3 months were excluded from the study. Institutional Review Board approval was obtained for this study and patients' consent was obtained from all women prior to inclusion in the study.

The diagnosis of PCOS was based on the 2003 Rotterdam ESHRE/ASRM criteria: (1) oligoovulation(cycle intervals > 35 days) and/or anovulation (absence of menstruation for 3 consecutive months); (2) clinical and/or biochemical signs of hyperandrogenism (patients presented with hirsute (Ferriman-Gallwey scale ≥ 6), acne or alopecia, and/or increased circulating levels of testosterone, T ≥ 2.8 nmol/L); (3) polycystic ovaries(12 or more follicles 2-9 mm in diameter and/or increased ovarian volume of more than 10 mL). All other aetiologies (congenital adrenal hyperplasia, androgen-secreting tumors, Cushing's syndrome) were excluded [8].

The control group consisted of 79 subjects with a mean age of 29.82 years (S.D. = 3.86). Controls were recruited by word of mouth and hospital advertisements calling for "healthy women". All controls underwent a brief history, physical exam and chemical examination of serum hormones, ensuring that all control women were non-hirsute and had regular menstrual cycles, normal androgen levels and had birthed at least one child.

For further analysis the PCOS patients were divided into two sub-groups three times. Firstly, PCOS IR group and PCOS without IR group, insulin resistance was judged by using the homeostatic model IR index (HOMA-IR), and 2.69 was selected as a cutoff point [9,10]. Secondly, the PCOS patients were divided into obese PCOS patients and lean PCOS patients, BMI < 25 kg/m^2 ^had been used as a cutoff point. Finally, PCOS with hyperandrogenism group and PCOS without hyperandrogenism: group, we assessed total testosterone, 2.8 nmol/L was used as a cutoff point.

### Blood and tissue DNA isolation

All subjects underwent a brief physical examination, including: height, weight, body mass index [BMI was calculated as follows: weight (kilograms)/height^2 ^(meters)] and blood sampling for hormonal measurement. Blood samples were obtained between days 2 and 3 of the menstrual cycle. In patients with amenorrhea, bleeding was induced by progestogens, with blood samples taken thereafter. If no bleeding occurred, blood samples were taken after validating the lack of an established pregnancy by a commercially available pregnancy test. Blood was taken from the antecubital vein after 12-hours of overnight fasting. Samples were immediately centrifuged, with the serum separated and frozen at -20°C until assayed.

Fasting insulin (FIN), follicle- stimulating hormone (FSH), luteinizing hormone (LH) and total testosterone (T) levels were determined by chemiluminescence- immunoassay (CLIA) and fasting glucose (FBG) levels were detected using the glucose oxidase method.

DNA was extracted from EDTA- collected peripheral whole blood with standard techniques following the manufacturer's instructions and stored at -20°C.

Fasting glucose and insulin values were also obtained for each of the subjects. The homeostasis model assessment (HOMA) was used to calculate indices of insulin resistance and insulin secretion for each patient [11]. Homeostasis model assessment index for IR (HOMA-IR) was calculated as follows: fasting insulin (mIU/l) × fasting glucose (mmol/L)/22.5.

### Polymerase chain reaction

Polymorphisms were analyzed by using PCR-SSP, at positions -607 and -137 in the promoter of the IL-18 gene [7]. For the position -607C/A- specific PCR, a common reverse primer 5'-TAACCTCATTCAGGACTTCC-3' and two sequence-specific forward primers 5'-GTTGCAGAAAGTGTAAAAATTATTA C-3' and 5'-GTTGCAGAAAGTGTAAAAATTATTAA-3' were used. An amplification product of 196 bp was detected. A control forward primer 5'-CTTTGC TATCATTCCAGGAA-3' was used to amplify a 301 bp fragment covering the polymorphic site as an internal positive amplification control. PCR reaction was performed in a final volume of 25 μL consisting of 2.5 μL 10 × PCR buffer, 0.5 μL dNTP, 1.5 μL genomic DNA and 0.5 μL Taq polymerase. One sequence-specific primer (for the C allele or A allele), the common reverse primer, and the internal position control primer were included in every reaction mixture at a concentration of 0.25 μL, respectively. Therefore, two PCR reactions were performed for every individual DNA.

Reactions were carried out in a Gene Amp PCR System 9600 (Perkin Elmer, USA) thermal cycler. Denaturation was performed for 2 min at 94°C, followed by 30 cycles at 94°C for 30 s, 57°C for 30 s, 72°C for 30 s and 72°C for 5 min. PCR products were visualized by 3% agarose gel electrophoresis. The molecular weight of PCR products was estimated using a 100-bp DNA ladder (Roche, Germany). PCR products were visualized and photographed under UV with an Alpha Imager 1220 v 5.5 Camera software

For the -137 genotyping, a common reverse primer 5'-AGGAGGGCAAAATGC ACTGG-3' and two sequence-specific forward primers 5'-CCCCAACTTTTACGG AAGAAAAG-3' and 5'-CCCCAACTTTTACGGAAGAAAAC-3' were used. An amplification product of 261 bp was detected. A control forward primer 5'-CCAATA GGACTGATTATTCCGCA-3' was used to amplify a 446 bp fragment covering the polymorphic site to serve as an internal positive amplification control. PCR reaction was performed in a final volume of 25 μL consisting of 2.5 μL 10 × PCR buffer, 0.5 μL dNTP, 1.5 μL genomic DNA and 0.5 μL Taq polymerase. The quantity of the control primer, the reverse primer, and sequence-specific primers was 0.25 μL, respectively. The PCR was run as follows: denaturation for 2 min at 94°C, followed by 30 cycles of 94°C for 30 s, 57°C for 30 s, 72°C for 30 s and 72°C for 5 min.

### Statistical analysis

The primary phenotype for genetic association analysis was the presence or absence of PCOS, given that such analysis utilizes all available subjects to maximize power. Secondary analyses included the insulin-related traits (fasting insulin, fasting glucose, HOMA-IR), adiposityrelated traits (BMI) and androgen-related traits (T), within PCOS.

The frequencies of genotypes and alleles in the promoter region of the IL-18 gene at positions -607 and -137 were calculated by counting. Data were analyzed with SPSS statistical software system (SPSS 11.5; SPSS Inc., Chicago, IL) and the Hardy-Weinberg equilibrium was determined by means of chi square testing. Statistical significance was assumed for P values less than 0.05.

## Results

### IL18 gene polymorphism

**Figure 1:**
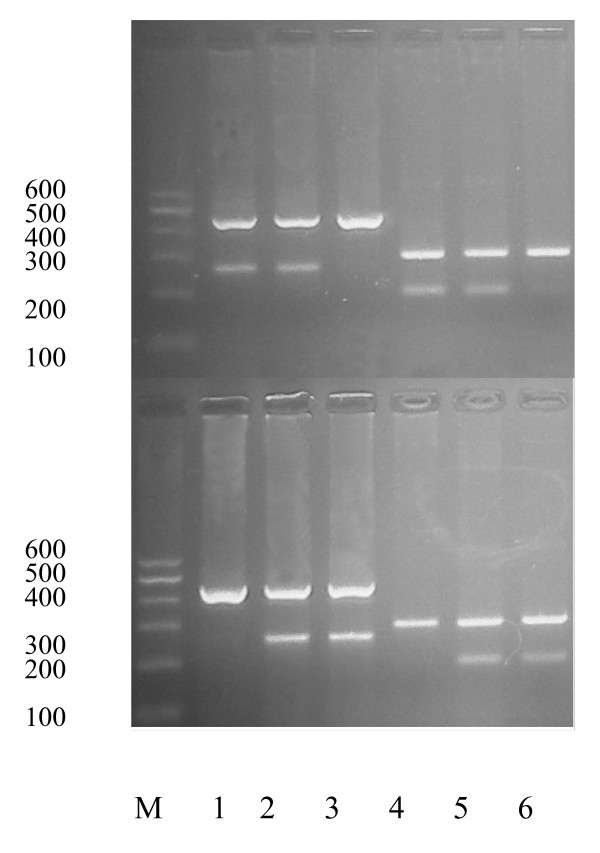
**Genotyping for the IL-18 position -137 and -607 polymorphisms**. M: DL600 DNA Marker; lanes 1-3: -137 GG, GC and CC; lanes 4-6: -607 CC, CA and AA.

Polymorphisms at positions -607 and -137 in the promoter of the IL-18 gene were analyzed by PCR-SSP. At every polymorphic site, a common reverse primer and two sequence-specific forward primers were used, resulting in two PCR reactions performed for every individual DNA. The specific PCR products from homozygous individuals showed one DNA segment, while heterozygous individuals exhibited two specific fragments, as expected. In total, 197 unrelated Chinese subjects were studied for IL-18 promoter polymorphisms. As shown in Figure 1, there were CC, CA and AA genotypes at position -607, and GG, GC and CC genotypes at position -137.

### Comparison of IL18 gene polymorphism between PCOS patients and controls

Genotype and allele frequencies for IL-18 polymorphisms are summarized in Table 1. The genotype frequencies were in agreement with the Hardy-Weinberg equilibrium (P > 0.1 for all analyses). For the -607 genotypes from the 118 PCOS patients, 39 had the CC type (33%), 61 the CA type (51.7%), and 18 the AA (15.3%). Of the 79 control subjects, 22 had the CC type (27.8%), 48 the CA type (60.8%), and 9 the AA type (11.4%). Regarding the -137 genotypes, 92 of the 118 PCOS patients had the GG type (78.0%), 25 the GC type (21.2%), and 1 the CC type (0.8%). Sixty-three of the 79 control subjects were type GG (79.7%) and 16 were GC (20.3%). No significant differences were observed in the genotype distribution or allele frequency between the PCOS and control groups.

**Table 1:** Comparison of IL18 gene promoter polymorphism between PCOS patients and controls

	PCOS N = 118(%)	controls n = 79(%)	*χ^2^*	P
Position -607 genotype				
CC	39(33.0)	22(27.8)	1.631	0.442
CA	61(51.7)	48(60.8)		
AA	18(15.3)	9(11.4)		
Alleles				
C	139(58.9)	92(58.2)	0.018	0.895
A	97(41.1)	66(41.8)		
Position -137 genotype				
GG	92(78.0)	63(79.7)	0.708	0.702
GC	25(21.2)	16(20.3)		
CC	1(0.8)	0		
Alleles				
G	209(88.6)	142(89.9)	0.168	0.682
C	27(11.4)	16(11.1)		

Based on genotypes of the IL-18 promoter polymorphisms, haplotype frequencies were estimated by the expectation maximization method. Four haplotypes of the IL-18 promoter at positions -607 and -137 were present in both patients and controls (haplotypes I, II, III and IV in Table 2). The frequencies of haplotypes I, II, III and IV in the PCOS patients were 57.0%, 0.6%, 30.7% and 11.7%, respectively. The frequencies of haplotypes I, II, III and IV in the controls were 28.2%, 0%, 31.6% and 10.2%, respectively. No significant differences were observed in the haplotype frequencies between the PCOS and the controls.

**Table 2:** Haplotype frequencies of two interleukin-18 bi-allellic polymorphisms in PCOS and controls

Haplotype	-607C/A	-137G/C	PCOS n (%)	controls n (%)	*χ^2^*	P
I	C	G	135(57.0)	92(58.2)	0.058	0.81
II	C	C	1(0.6)	0(0)	0.406	0.52
III	A	G	72(30.7)	50(31.6)	0.039	0.84
IV	A	C	28(11.7)	16(10.2)	0.228	0.63
Total			236	158		

### Comparison of IL18 gene polymorphism between PCOS patients with IRand without IR

For further analysis of the relationship between IL-18 gene promoter polymorphisms and PCOS IR. Insulin resistance was judged by using the homeostatic model IR index (HOMA-IR), and 2.69 was selected as a cutoff point [9,10]. The PCOS patients were divided into two sub-groups: PCOS IR: HOMA-IR ≥ 2.69; PCOS without IR: HOMA-IR < 2.69. Genotype and allele frequencies for IL-18 polymorphisms are summarized in Table 3. There were no significant differences in the genotype frequencies between the two sub-groups. As for the -137 allele frequencies, G allele frequency was 93.5% and C allele frequency was 6.5% in the PCOS IR group. In the PCOS without IR group, the G allele frequency was 85.4% and C allele frequency was 14.6%. There was indeed a significant difference in the allele frequency between the two sub-groups (P = 0.048). Four haplotypes of the IL-18 promoter at positions -607 and -137 were present in the two sub-groups; they are summarized in Table 4. There were no significant differences in the haplotype frequencies between the PCOS IR and PCOS without IR groups.

**Table 3:** Comparison of IL18 gene promoter polymorphism between PCOS patients with IR and without IR

	PCOS IR n = 46 (%)	PCOS without IR n=72 (%)	*χ^2^*	P
Position -607 genotype				
CC	19(41.3)	20(27.7)	2.328	0.312
CA	21(45.7)	40(55.6)		
AA	6(13.0)	12(16.7)		
Alleles				
C	59(64.1)	80(55.6)	1.705	0.192
A	33(35.9)	64(44.4)		
Position -137 genotype				
GG	40(87.0)	52(72.2)	3.780	0.151
GC	6(13.0)	19(26.4)		
CC	0(0)	1(1.4)		
Alleles				
G	86(93.5)	123(85.4)	3.601	0.048
C	6(6.5)	21(14.6)		

**Table 4:** Haplotype frequencies of two interleukin-18 bi-allelic polymorphisms in PCOS patients with IR and without IR

Haplotype	-607C/A	-137G/C	PCOS IR n (%)	PCOS without IR n (%)	*χ^2^*	P
I	C	G	59(64.1)	79(54.7)	2.035	0.15
II	C	C	0(0)	3(2.2)	1.293	0.26
III	A	G	27(29.4)	45(31.4)	0.108	0.74
IV	A	C	6(6.5)	17(11.7)	1.72	0.19
Total			92	144		

### Comparison of IL18 gene polymorphism between obese and lean PCOS women

For further analysis of the relationship between the IL-18 gene promoter polymorphisms and PCOS IR, the PCOS patients were again divided into two sub-groups: obese PCOS: BMI ≥ 25 kg/m^2^; lean PCOS: BMI < 25 kg/m^2^. Genotype and allele frequencies for IL-18 polymorphisms are summarized in Table 5; four haplotypes of the IL-18 promoter at positions -607 and -137 were present in the two sub-groups, which are summarized in Table 6. There were no significant differences in the genotype distribution, allele frequencies, or haplotype frequencies between obese and lean PCOS women.

**Table 5:** Comparison of IL18 gene promoter polymorphism between obese PCOS women and lean PCOS women

	Obese PCOS n=54(%)	Lean PCOS n=64(%)	*χ^2^*	P
Position -607 genotype				
CC	9(14.0)	9(16.7)	0.510	0.775
CA	26(54.7)	35(48.1)		
AA	19(31.3)	20(35.2)		
Alleles				
C	44(40.7)	53(41.4)	0.011	0.918
A	64(59.3)	75(58.6)		
Position -137 genotype				
GG	42(77.7)	50(78.1)	1.217	0.544
GC	11(20.4)	14(21.9)		
CC	1(1.9)	0		
Alleles				
G	95(88.0)	114(89.1)	0.070	0.791
C	13(12.0)	14(10.9)		

**Table 6:** Haplotype frequencies of two interleukin-18 bi-allelic polymorphisms in obese PCOS women and lean PCOS women

Haplotype	-607C/A	-137G/C	Obese PCOS n (%)	Lean PCOS n (%)	*χ^2^*	P
I	C	G	64(59.3)	73(57.3)	0.088	0.77
II	C	C	0(0)	2(1.3)	0.749	0.39
III	A	G	31(28.7)	41(31.7)	0.252	0.62
IV	A	C	13(12.0)	12(9.7)	0.338	0.56
Total			108	128		

### Comparison of IL18 gene polymorphism between PCOS patients with and without hyperandrogenism

For further analysis of the relationship between the IL-18 gene promoter polymorphisms and PCOS IR, the PCOS patients were again divided into two sub-groups: PCOS with hyperandrogenism: T ≥ 2.8 nmol/L; PCOS without hyperandrogenism: T < 2.8 nmol/L. Genotype and allele frequencies for IL-18 polymorphisms are summarized in Table 7; four haplotypes of the IL-18 promoter at positions -607 and -137 were present in the two sub-groups, and are summarized in Table 8. There were no significant differences in the genotype distribution, allele frequencies, or haplotype frequencies between PCOS subjects with and without hyperandrogenism.

**Table 7:** Comparison of IL18 gene promoter polymorphism between PCOS patients with and without hyperandrogenism

	PCOS HA n=50(%)	PCOS without HA n=68(%)	*χ^2^*	P
Position -607 genotype				
CC	21(42.0)	18(26.5)	3.186	0.203
CA	22(44.0)	39(57.4)		
AA	7(14.0)	11(16.1)		
Alleles				
C	64(64.0)	75(55.1)	1.866	0.172
A	36(36.0)	61(44.9)		
Position -137 genotype				
GG	40(80.0)	52(76.5)	0.839	0.657
GC	10(20.0)	15(22.0)		
CC	0(0.0)	1(1.5)		
Alleles				
G	90(90.0)	119(87.5)	0.355	0.551
C	10(10.0)	17(12.5)		

**Table 8:** Haplotype frequencies of two interleukin-18 bi-allelic polymorphisms in PCOS patients with and without hyperandrogenism

Haplotype	-607C/A	-137G/C	PCOS HA n (%)	PCOS without HA n (%)	*χ^2^*	P
I	C	G	61(60.6)	75(55.2)	0.708	0.400
II	C	C	1(1.4)	0(0)	1.294	0.26
III	A	G	28(28.4)	46(33.8)	0.793	0.37
IV	A	C	10(9.6)	15(11.0)	.121	0.73
Total			100	136		

## Discussion

IL-18 is a proinflammatory cytokine that induces the production of TNF-α [12], IL-6 [13], and C-Reactive Protein (CRP) [14]. Like IL-6 and CRP, IL-18 is considered a strong risk marker for cardiovascular death [15]. Women with PCOS have chronic low-level inflammation [16,17], often associated with obesity and a subsequent increased risk for type 2 diabetes [18-20]. Recently, study has shown that PCOS induces an increase in serum IL-18 levels, which are also associated with several indexes of global and visceral adiposity, and with insulin IR [21]. It has been suggested that IL-18 may be a contributing factor linking inflammation and IR in PCOS women [22].

The human IL-18 gene is located on chromosome 11q22.2-q22.3; it is composed of six exons and five introns [23]. Kruse et al. [24] and Sugiura et al. [25] described three SNPs at positions -656G/T, -607C/A and -137G/C in the promoter of the first exon of the IL-18 gene. A change from C to A at position -607 disrupts a potential cAMP-responsive element-binding protein binding site; a change at position -137 from G to C changes the H4TF-1 nuclear factor binding site. Cloning and gene expression analysis demonstrated that two SNPs of the IL-18 promoter at positions -607 and -137 were suggested to cause the differences in transcription factor binding. They may also have an impact on IL-18 gene activity and also to interferon-γ (IFN). Potentially, the G/C polymorphisms at position -137 could play a major role in the expression of IL-18.

These promoter SNPs have also been implicated as susceptibility loci for various diseases, including type 1 diabetes [25,26], rheumatoid arthritis [27,28], sarcoidosis [29], atopic eczema [30], adult-onset Still's disease [24], and seasonal allergic rhinitis [23]. Kretowski et al. [25] genotyped 201 case and 194 unrelated control subjects from Poland, reporting that the C allele at -137 (G-C) was associated with type 1 diabetes (odds ratio [OR] 1.6; *P *= 0.002). At -607, case subjects reported an increase in the proportion of the C/A genotype relative to control subjects (64.2% case and 56.7% control subjects), while a decrease was seen in the proportion of the A/A genotype in case subjects (0% case and 5.7% control subjects).

In the present study, two polymorphisms in the promoter regions of the IL-18 gene were identified. The association between these polymorphisms and PCOS were demonstrated. The results showed no significant differences in the distribution of genotypes, allelic or haplotype frequencies for polymorphisms of the IL-18 gene between PCOS patients and control subjects. IL-18 gene polymorphisms had no direct relationship with the pathogenesis of PCOS.

Further analysis of the relationship between IL-18 gene promoter polymorphisms and the existence of PCOS IR showed that C allele frequency at position -137 in the IL-18 gene promoter was closely associated with PCOS IR (chi2 = 3.601, P = 0.048 < 0.05). Carriage of the C allele at position -137 in the promoter of the IL-18 gene may play a protective role from the development of PCOS IR.

The relationship between IL-18 gene promoter polymorphism and PCOS obesity and hyperandrogenism were also studied; the results showed that the distribution of genotypes, allelic or haplotype frequencies for polymorphisms of the IL-18 gene, have no differences. It illustrated that the role of IL-18 gene promoter polymorphism had no relationship with obesity and hyperandrogenism of PCOS patients.

This was the first research exploring the role of IL-18 gene promoter polymorphism in the etiology of PCOS and its relation with IR and obesity.But we study only two site of IL-18 gene polymorphism, we will continue our work in the future. Moreover, the samples of this study was not large enough, the real roles of IL-18 in the pathogenesis of developing PCOS should be further investigated by large population-based studies.

## Conclusions

In summary, IL-18 gene polymorphisms had no direct relationship with the pathogenesis of PCOS. Our results suggest that carriage of the C allele at position -137 in the promoter of the IL-18 gene may play a protective role from the development of PCOS IR.

## Competing interests

The authors declare that they have no competing interests.

## Authors' contributions

YY, JQ and MZ-L developed the concept and designed the study. YY and JQ participated in the study execution, analysed and interpreted the data and drafted the manuscript. JQ and MZ-L revised the manuscript for intellectual content. All authors read and approved the final manuscript.
